# High diagnostic performance of independent alpha-synuclein seed amplification assays for detection of early Parkinson’s disease

**DOI:** 10.1186/s40478-021-01282-8

**Published:** 2021-11-06

**Authors:** Marco J. Russo, Christina D. Orru, Luis Concha-Marambio, Simone Giaisi, Bradley R. Groveman, Carly M. Farris, Bret Holguin, Andrew G. Hughson, David-Erick LaFontant, Chelsea Caspell-Garcia, Christopher S. Coffey, Jennifer Mollon, Samantha J. Hutten, Kalpana Merchant, Roland G. Heym, Claudio Soto, Byron Caughey, Un Jung Kang

**Affiliations:** 1 The Marlene and Paolo Fresco Institute for Parkinson’s & Movement Disorders, Department of Neurology, Department of Neuroscience and Physiology, Neuroscience Institute, The Parekh Center for Interdisciplinary Neurology, NYU Grossman School of Medicine, New York, NY USA; 2 Laboratory of Persistent Viral Diseases, Rocky Mountain Laboratories, National Institute of Allergy and Infectious Diseases, National Institutes of Health, Hamilton, MT USA; 3 R&D Unit, Amprion Inc., San Diego, CA USA; 4 AbbVie Deutschland GmbH & Co. KG, Ludwigshafen, Germany; 5 Department of Biostatistics, College of Public Health, University of Iowa, Iowa City, IA USA; 6 Michael J. Fox Foundation for Parkinson’s Research, New York, NY USA; 7 Northwestern University Feinberg School of Medicine, Chicago, IL USA; 8 Mitchell Center for Alzheimer’s Disease and Related Brain Disorders, Department of Neurology, University of Texas Houston Medical School, Houston, TX USA

**Keywords:** Seed amplification assay, Alpha-synuclein, Parkinson’s disease, RT-QuIC, PMCA, Synucleinopathy

## Abstract

Alpha-synuclein seed amplification assays (αSyn-SAAs) are promising diagnostic tools for Parkinson’s disease (PD) and related synucleinopathies. They enable detection of seeding-competent alpha-synuclein aggregates in living patients and have shown high diagnostic accuracy in several PD and other synucleinopathy patient cohorts. However, there has been confusion about αSyn-SAAs for their methodology, nomenclature, and relative accuracies when performed by various laboratories. We compared αSyn-SAA results obtained from three independent laboratories to evaluate reproducibility across methodological variations. We utilized the Parkinson’s Progression Markers Initiative (PPMI) cohort, with DATSCAN data available for comparison, since clinical diagnosis of early de novo PD is critical for neuroprotective trials, which often use dopamine transporter imaging to enrich their cohorts. Blinded cerebrospinal fluid (CSF) samples for a randomly selected subset of PPMI subjects (30 PD, 30 HC, and 20 SWEDD), from both baseline and year 3 collections for the PD and HC groups (140 total CSF samples) were analyzed in parallel by each lab according to their own established and optimized αSyn-SAA protocols. The αSyn-SAA results were remarkably similar across laboratories, displaying high diagnostic performance (sensitivity ranging from 86 to 96% and specificity from 93 to 100%). The assays were also concordant for samples with results that differed from clinical diagnosis, including 2 PD patients determined to be clinically inconsistent with PD at later time points. All three assays also detected 2 SWEDD subjects as αSyn-SAA positive who later developed PD with abnormal DAT-SPECT. These multi-laboratory results confirm the reproducibility and value of αSyn-SAA as diagnostic tools, illustrate reproducibility of the assay in expert hands, and suggest that αSyn-SAA has potential to provide earlier diagnosis with comparable or superior accuracy to existing methods.

**Supplementary Information:**

The online version contains supplementary material available at 10.1186/s40478-021-01282-8.

## Introduction

The pathologic signature of Parkinson disease (PD) is accumulation of misfolded, aggregated forms of α-synuclein (αSyn) throughout the nervous system. Deposition of αSyn and neuronal injury occur many years before the canonical motor signs emerge [1, 2]. PD is diagnosed primarily through clinical assessment, which can be supported by striatal dopamine-transporter imaging (*e.g.* DAT-SPECT). Both clinical and radiologic diagnostic accuracy improve with progression of disease, and require that some degree of nigrostriatal dysfunction is already manifest [3]. During early stages of disease, these assessments can fail to distinguish classic PD from other synucleinopathies such as multiple system atrophy (MSA), and from non-αSyn-driven forms of parkinsonism, such as progressive supranuclear palsy (PSP) or corticobasal degeneration (CBD). Ultrasensitive diagnostic assays are now available that enable detection and classification of synucleinopathies based on the presence of misfolded αSyn forms in CSF that ‘seed’, or induce, aggregation of native αSyn. These seed amplification assays (SAAs) provide more direct etiologic diagnosis and can reveal αSyn pathology that is independent of nigrostriatal injury.

SAAs were originally developed for antemortem detection of prions (PrP^Sc^), the pathologic form of the normally occurring prion protein (PrP^C^), and the pathogenic agent in diseases such as Creutzfeldt-Jakob disease and kuru [4]. SAAs are based on the tendency of PrP^Sc^ seeds to induce native proteins to shift to conformations that favor polymerization. They have been adapted to detect αSyn seeds and have been reported under the names real-time quaking-induced conversion (RT-QuIC) [5–7], protein misfolding cyclic amplification (PMCA) [8, 9], seeding aggregation assay (SAA) [7], or seed amplification assay (SAA) [10]. The operational principles of these are very similar, and so we propose to unify the nomenclature for RT-QuIC and PMCA under one name—seed amplification assay (SAA)—to better capture the nature of the technology and avoid confusion with the previous prion-based amplification assays.

For αSyn-SAA, biospecimens are added to a reaction chamber containing excess of recombinant αSyn (rec-αSyn). Any αSyn seeds in the specimen will convert rec-αSyn to misfolded forms and promote fibrillization. Mechanical disruption of nascent aggregates generates fragments to seed further polymerization, which effectively amplifies the biomarker to detectable levels. The in vitro generated αSyn fibrils are rich in β-sheets, enabling detection by the amyloid-specific dye Thioflavin T (ThT). αSyn-SAAs are capable of detecting and amplifying αSyn seeds from multiple biospecimens, including brain [11–13], cerebrospinal fluid (CSF) [6–10, 13–16], skin [17–19], olfactory mucosa [20, 21], submandibular gland [22], and gut [23]. Moreover, they have demonstrated accurate detection of αSyn seeds in clinically and pathologically validated cohorts of PD patients [6, 7, 9, 10, 13, 17, 18, 24]. Interestingly, αSyn-SAAs have also been shown to distinguish αSyn aggregates from different synucleinopathies, such as PD and MSA [25, 26]. However, performing SAAs is not trivial, and differences in methodologies under various names applied in different cohorts have generated confusion as to their nature and relative accuracies.

We performed a blind comparative study of αSyn-SAA by three laboratories, two of whom have pioneered these assays and a third industry lab with their own newly instituted version. Each lab performed αSyn-SAA according to their preferred methods, without coordination of procedures, reagents, or results. We utilized CSF from the PPMI cohort of PD and HC since diagnosis of early de novo PD can be challenging, and yet critical for clinical trials of neuroprotective agents. DAT imaging is often used to enrich the study population. We also included a subset of subjects designated as scans without evidence of dopaminergic deficit (SWEDD), with parkinsonian motor signs, but ostensibly normal DAT-SPECT imaging. Many of these subjects are likely to have alternative diagnoses (*e.g.* essential tremor), but it is suspected that a subset has *bona fide* PD in an early form without appreciable deficit with dopamine transporter imaging [27, 28]. We report the reproducibility of αSyn-SAAs across practitioners and differences in methods or analysis, and further validate the diagnostic potential of SAA for well-characterized early de novo PD.

## Materials and methods

### CSF sample collection

All CSF samples and clinical data were collected according to the Parkinson’s Progression Markers Initiative (PPMI) study protocol. Per PPMI inclusion criteria, PD subjects were enrolled within 2 years of diagnosis, not treated with PD medications at time of enrollment, Hoehn and Yahr stage 1–2, had abnormal DAT-SPECT demonstrating striatal dopaminergic denervation, and had two of the following: resting tremor, bradykinesia, rigidity (required to have either resting tremor or bradykinesia); or either asymmetric resting tremor or asymmetric bradykinesia. Subjects with scans without evidence of dopamine deficit (SWEDD) were enrolled with PD inclusion criteria, with major exception that initial DAT-SPECT did not show evidence of decreased striatal radioligand uptake. Healthy controls (HC) were age- and gender-matched healthy persons without known neurologic signs or symptoms.

**Fig. 1:**
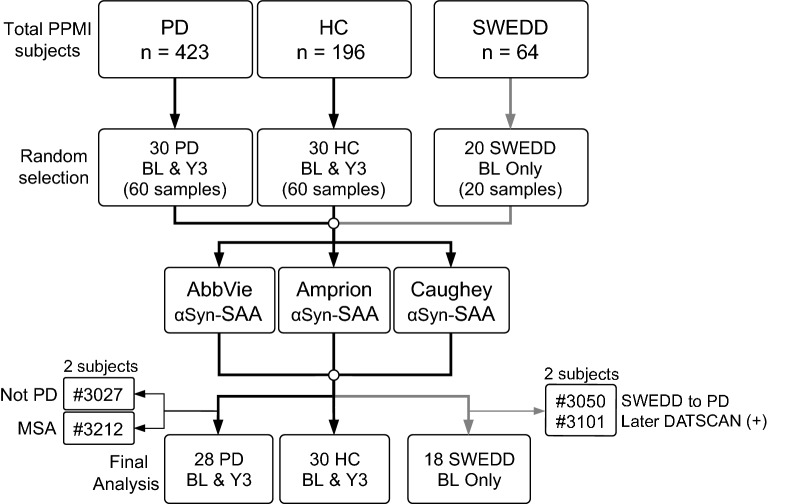
Experimental design. PD, HC, and SWEDD samples were randomly selected from available PPMI subjects as a pilot study. Aliquots from each subject were distributed to three independent laboratories for αSyn-SAA according to proprietary methods without procedural coordination or communication. αSyn-SAA was performed and initially analyzed in blinded manner with respect to diagnosis. After completion of assays, 2 PD and 2 SWEDD subjects were removed from further analysis due to interval changes in consensus diagnosis

From the total pool of PPMI subjects (423 PD, 196 HC, 64 SWEDD) with available CSF, 30 PD subjects, 30 HC subjects, and 20 SWEDD subjects were randomly selected for this study (Fig. 1). αSyn-SAAs were performed by three labs according to methods described below. After samples were analyzed, ongoing review by PPMI led to revision of diagnoses of 4 patients through the consensus decision of a clinical review committee. These subjects were removed from final analysis of assay performance. Complete demographic data for the tested cohort within context of the complete PPMI cohort is provided in Additional file 1: Table S1. Average age of the PD αSyn-SAA cohort was 62.1 ± 9.3 years old, with average H&Y stage 1.5 ± 0.5, and average baseline UPDRS Part 3 / total score 20.5 ± 8.6 / 33.9 ± 13.9; with 19 males and 9 females. Healthy controls were aged 63.8 ± 10.6 years, with 18 males and 12 females. The demographic, clinical, and biomarker features of selected cohort were representative of the entire PPMI cohort. Age and gender were not significantly different between HC and PD selected cohorts. The SWEDD αSyn-SAA cohort average age is 59.6 ± 10.6, with baseline UPDRS Part 3 / total scores of 12.1 ± 9.4 / 28.1 ± 16.1 and H&Y 1.3 ± 0.5, with 12 males and 6 females. Age, gender, disease duration, and ages of disease onset and diagnosis were similar for PD and SWEDD groups (Additional file 1: Table S1). CSF samples at baseline and at year 3 (Y3, corresponding to PPMI Visit 08) were included for PD and HC subjects.

Detailed information about inclusion criteria, informed consent, demographic data, and study design can be found at www.ppmi-info.org/study-design/research-documents-and-sops/archive-of-study-protocols. Assay data from all three groups are available in the PPMI LONI database (AbbVie: project #173; Amprion: project #155; Caughey: project #172).

Additional clinical and biomarker data from PPMI were used for further analysis with αSyn-SAA kinetic parameters. These include the MDS Unified Parkinson’s Disease Rating Scale (UPDRS, only ‘off’ scores were included), Montreal Cognitive Assessment (MoCA), rapid eye movement (REM)-sleep behavior disorder questionnaire (RBDQ), University of Pennsylvania Smell Identification Test (UPSIT), Scales for Outcomes in Parkinson’s-Autonomic Dysfunction (SCOPA-AUT), DAT-SPECT specific binding ratio (SBR), amyloid-β (Aβ), tau, and neurofilament light chain (NfL).

### αSyn seed amplification assays

Performance of all assays and initial analyses were blinded to patient indicators and diagnostic categories. Use of FLUOstar Omega (BMG) fluorometers, and use of ThT (10 μM) as fluorescent amyloid indicator with 450 ± 10 nm excitation and 480 ± 10 nm emission, were common to all three assays. Each laboratory performed αSyn-SAA according to methods outlined below, with side-by-side method summary in Table 1.

**Table 1:** Summary of αSyn-SAA reagents and protocols for each laboratory

	AbbVie	Amprion	Caughey
Monomeric αSyn	0.1 mg/ml recombinant human αSyn (w/o tag)	0.3 mg/ml recombinant αSyn (C-term His)	0.1 mg/ml recombinant K23Q αSyn (N-term His)
CSF	5% v/v	20% v/v	15% v/v
Reaction volume	100 μl	200 μl	100 μl
Reaction duration	70 h	150 h	48 h
Reaction mixture	100 mM NaH_2_PO_4_ (pH to 8.2 w/ NaOH)	500 mM NaCl 100 mM PIPES (pH to 6.5 w/ NaOH)	170 mM NaCl 40 mM NaH_2_PO_4_/ Na_2_HPO_4_ (pH to 8.0 w/ Na_2_HPO_4_) 0.0015% SDS
Temperature	33 °C	37 °C	42 °C
Beads	25 ± 3 mg of 0.10-mm zirconia/silica beads	Single 3/32-inch Si_3_N_4_ bead	0.8-mm silica beads (6)
Shaking protocol	200 rpm shaking for 60 s and 14 min rest	700 rpm shaking for 60 s every 30 min	400 rpm shaking for 60 s every 60 s
No. Replicates	8	3	4
Threshold (RFU)	3500	Probabilistic algorithm based on assay kinetic parameters	10% plate maximum fluorescence
Positive	≥ 50% positive replicates (4–8)	All 3 replicates positive by algorithm	> 25% replicates positive
Negative	< 50% positive replicates (0–3)	0–1 positive replicates	0 positive replicates
Inconclusive	n/a	2 positive replicates	if 25% replicates positive, retested

Abbreviations: RFU, relative fluorescence units; rpm, rotations per minute; PIPES, piperazine-N,N’-bis(2-ethanesulfonic acid); SDS, sodium dodecyl sulfate; ThT, thioflavin T

### AbbVie αSyn-SAA method

Human recombinant full-length (1–140 aa) αSyn (rec-αSyn) without tag was expressed in *E. coli* and purified using standard purification methods. αSyn from one single purification batch was used throughout this study. αSyn-SAA reaction buffer was composed of 100 mM monosodium phosphate buffer (pH 8.2, adjusted with NaOH), 10 µM ThT and 0.1 mg/ml rec-αSyn. Each well of a black 96-well plate (Greiner Bio-One, Germany) contained 95 µl reaction buffer and 25 ± 3 mg of 0.1 mm zirconia/silica beads (Roth, Karlsruhe, Germany). Reactions were seeded with 5 µl of undiluted CSF samples yielding final reaction volume of 100 µl per well. Plates were sealed with a plate sealer film (Fisher Scientific Ltd, UK) and incubated in the plate reader at 33 °C for 70 h with shake/rest cycles: 1 min double orbital shaking at 200 rpm, 14 min rest. ThT fluorescence measurements were taken every 15 min. Each CSF sample was measured in two independent assay runs with 4 technical replicates each. In total, 16 plates were measured.

Before further analysis, each aggregation curve was baseline corrected by subtracting the mean of the relative fluorescence units (RFU) measured between 2 and 5 h from all data points of the curve. A threshold of 3500 RFU was used to determine the time to threshold (TTT), *i.e.* time when the curve reaches this threshold. If threshold was not reached during the 70 h runtime of the assay, the TTT was set to 70 h. Curves that reached threshold were defined as positive replicates. A sample was defined as αSyn-SAA positive if 50% or more of the replicates were positive (4 to 8 replicates) and as negative if less than 50% of the replicates were positive (0 to 3 replicates). Maximum fluorescence (*F*_max_) was determined as highest RFU value recorded during the 70 h assay runtime. Area under curve (AUC) was calculated between 0 and 70 h.

### Amprion αSyn-SAA method

The Amprion αSyn-SAA is based on the protein misfolding cyclic amplification (PMCA) platform developed by Soto and colleagues [8, 9] and has been described previously [10]. The 200-μl reaction mixture included 0.3 mg/mL rec-αSyn, 0.5 M NaCl, 100 mM PIPES (pH adjusted to 6.5 with NaOH), and 20% v/v CSF. Rec-αSyn was expressed with C-terminal His-tag in *E. coli* BL21(DE3) and purified using immobilized metal affinity chromatography (IMAC). All samples were analyzed with a single batch of substrate. One 3/32″ Si_3_N_4_ bead (Tsubaki Nakashima) was added per well using a house-made bead dispenser. Beads were blocked with 1% BSA in 100 mM PIPES pH 6.5 for 1 h and washed twice with 100 mM PIPES pH 6.5. Samples were run in 3 technical replicates within 96-well plates, using plate reader coupled to a robotic arm inside an incubator set to 37 °C. Each plate was shaken for 1 min every 29 min, and fluorescence was measured after each cycle for 150 h. In these assay conditions, CSF samples containing αSyn aggregates typically show a classic amyloid aggregation curve within 100 h. Kinetic values obtained from aggregation curves include *F*_max_ (highest raw fluorescence from each well), T_50_ (time to reach 50% of the *F*_max_), and TTT (time to reach a 5,000 RFU threshold). Using data from a training set of ~ 1,000 samples, a proprietary probabilistic algorithm was developed to determine whether each well was positive or negative. Based on these results, if all 3 replicates from a given sample were positive, the sample is positive. If only 0–1 replicates are positive by the algorithm, the sample is negative. If 2 of 3 replicates are positive, the sample was labeled inconclusive. A second-level criterion within the algorithm compared average *F*_max_ of all 3 wells from inconclusive samples, and samples with highly variable or low *F*_max_ were called negative.

### Caughey αSyn-SAA method

αSyn-SAA performed by the Caughey lab is also referred to as real-time quaking induced conversion (RT-QuIC) assay and methods are similar to those described previously [13, 29]. CSF samples were thawed and 15 μl immediately added to the seed reaction mixture (85 μl). Reaction mixture contained (final concentration in 100 μl): 40 mM phosphate buffer (prepared by mixing monosodium and disodium phosphates and finalizing pH to 8.0 using disodium phosphate), 170 mM NaCl, 0.1 mg/ml K23Q rec-αSyn, 10 μM ThT, and 0.0015% sodium dodecyl sulfate (SDS). All solutions and recombinant K23Q αSyn substrate batches were tested for adequate performance prior to use. Assay was performed with cycles of 1 min shaking (400 rpm double orbital) and 1 min rest throughout the indicated incubation time. ThT fluorescence was measured every 45 min. Each experimental plate was analyzed separately to account for differences between runs and between plate readers.

A 5-h baseline, consisting of the window from ~ 2–7 h was subtracted from each curve. Maximum ThT value on the plate within 48-h was then used to normalize all curves from 0 (lowest value for an individual curve) to 100% (maximum value on the plate). A 10% threshold was set as criterion for a well to be considered positive. The maximum value for each well was recorded as a percentage and then given a positive/negative determination based on the 10% threshold. Samples were analyzed in 4 technical replicates. For a sample to be considered positive, > 25% of the replicate wells needed to be positive. Samples with 25% of the replicate wells positive were considered inconclusive and re-tested in 4 technical replicates. If upon repeat they had ≤ 25% wells positive they were considered negative and marked as “negative (inconclusive)”. Samples with no positive wells were considered negative. TTT was calculated as the amount of time needed for a well to cross that 10% threshold. T_50_ was calculated by dividing the maximum fluorescence value for a well by 2 and then finding the time value closest to when that fluorescence value was reached. AUC, along with each of the other described parameters above (except for T_50_ which was calculated using Microsoft excel), was calculated using the OMEGA MARS software V3.32 Build 5. The same baseline subtracted, scaled curves were used to calculate area under the curve. All ThT negative wells were reported as “NA”.

### Inconclusive results

**Fig. 2:**
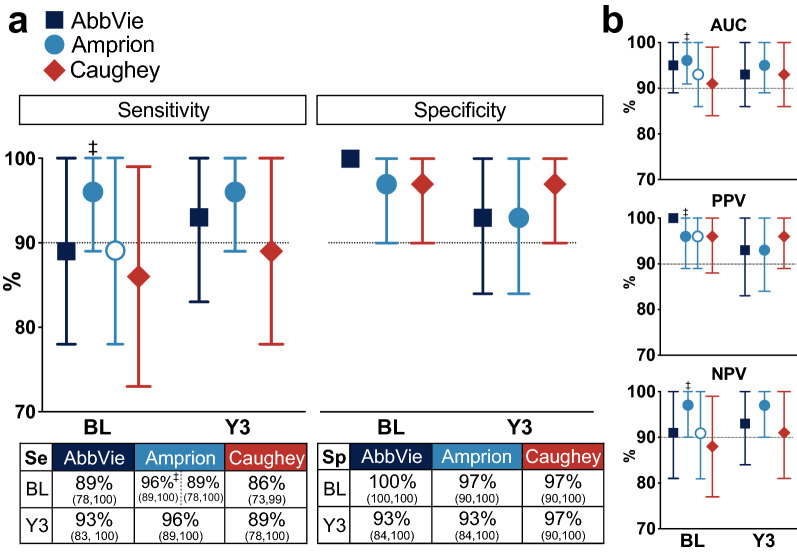
Seed amplification assay diagnostic performance for early PD. **a** Plots of sensitivity (left) and specificity (right) for SAA by each of the three laboratories (AbbVie, Amprion, and Caughey) at BL and Y3. Values (percentages) are provided for sensitivity and specificity in the tables below the respective plots. Calculations for Amprion αSyn-SAA are reported when 2 inconclusive results are excluded (filled circles), or when they are included as negative results (open circles). Amprion αSyn-SAA sensitivity is 96% if two inconclusive results (only 2/3 positive replicates) are excluded from analysis (filled circles), but 89% if these are treated as negative (not meeting 3/3 criterion for positive result). Specificity calculation and year 3 results were not affected. **b** Plots for area under the curve (AUC), positive predictive value (PPV), and negative predictive value (NPV). ‡ Inconclusive results (2 samples at baseline) not included in calculation

Among the data sets, there are differences in handling of inconclusive results. The algorithm developed by Amprion is based on 3 technical replicates and has ternary output (positive, negative, and inconclusive). Within the original MJFF research plan, Amprion allocated some volume of the sample to be tested by an earlier version of their αSyn-SAA [10], and there was not sufficient material to run repeated replicates of this assay in order to retest inconclusive results. We therefore report calculations for Amprion data both with inconclusive results excluded, and with inconclusive samples included as negative results (2 of 3 replicates positive) as a hypothetical worst-case scenario for calculating sensitivity—with both calculations reported in Fig. 2. Amprion’s experience with inconclusive results is that most become conclusive upon re-testing. The Caughey group ran 4 replicates initially, with ≥ 2 positive replicates indicating positive result. Any sets with only 1 positive replicate (25%) were repeated, again as 4 replicates, and this invariably produced unequivocally negative results (zero positive). Higher numbers of replicates probably facilitates the generation of conclusive results. AbbVie ran 8 replicates from each initial CSF sample, with ≥ 4 positive replicates indicating positive sample, and resulting in unambiguous results across samples.

### End-Point dilution analysis

End-point dilution analysis was performed on a subset of PD cohort samples with ≥ 75% positive αSyn-SAA replicates. All end-point dilution assays were performed by the Caughey laboratory similarly to protocols previously described [13, 30]. CSF was serially diluted 1:2 in pooled CSF from healthy samples with negative SAA, purchased from Innovative Research, Inc. (Michigan, USA). These dilutions underwent aggregation and shaking as per the protocol described above. The Spearman-Kärber method was used to calculate the sample dilution containing the amount of seed giving 50% positive replicate reactions, *i.e.* the 50% seeding dose or SD50 [30]. This dilution was then used to calculate the SD50 per volume of neat CSF (in this case, 15 µL of the original sample).

### Statistics and additional data analysis

Python 3.8, Pandas 1.2.4/Seaborn 0.11.1, Prism 9.1, and SAS 9.4 were used for statistical analysis and data presentation. Sensitivity and specificity were calculated from a non-parameterized receiver operator characteristic (ROC) calculation for each group with 95% confidence intervals. Correlation coefficients for all pairings were calculated using non-parametric rank-order methods (Spearman), producing a correlation coefficient (Spearman r) and p-values for null hypothesis that no monotonic relationship in the ranks exists. These p-values for ranks should be interpreted with caution, as we did not apply correction for multiple comparisons (i.e. Bonferroni correction). We employed linear regression to assess the association between SD50 and CSF NfL at baseline, adjusting for age. Although the model assumptions were met, due to a small number of samples, the linear regression results should be interpreted with caution.

## Results

### Diagnostic performance for PD

120 CSF samples were analyzed, from 30 PD and 30 HC subjects (Fig. 1). PD and HC samples were collected at BL and Y3. αSyn-SAA by all three groups demonstrated considerable accuracy in distinguishing PDs from HCs, and results were highly concordant. Performance data are summarized in Fig. 2. Sensitivity and specificity are expressed as percentages, with 95%-confidence interval in parentheses. For samples obtained at baseline/enrollment, the AbbVie αSyn-SAA was 89 (78, 100)% sensitive and 100 (100, 100)% specific. The Amprion αSyn-SAA was 96 (89, 100)% sensitive (when 2 inconclusive results—with only 2 of 3 replicates positive—were excluded from the calculation, and 89 [78, 100]% sensitive when these samples were considered negative) and 97 (90, 100)% specific. The Caughey αSyn-SAA was 86 (73, 99)% sensitive and 97 (90, 100)% specific. There was trend toward slightly improved sensitivity from BL to Y3 in results from two groups (AbbVie 89% → 93% and Caughey 86% → 89%). Raw numbers of positive/negative/inconclusive samples for each group are provided in Additional file 1: Fig. S1.

During this study, there were 2 subjects whose diagnoses were revised from the original PD designation applied at baseline (Fig. 1). These subjects were removed from calculations of sensitivity and specificity, as the intent was to validate these assays against the best available clinical diagnosis. One of these subjects (#3212) rapidly progressed and died during the PPMI study. Autopsy revealed widespread glial cytoplasmic inclusions, brainstem Lewy body disease, and bilateral gliosis of the lateral putamen, consistent with multiple system atrophy-parkinsonian type (MSA-P). The other subject was determined to have an uncertain diagnosis but not PD through consensus review by an independent PPMI committee. Interestingly, αSyn-SAA results by all three groups were negative for both of these subjects, at both BL and Y3.

There were 7 PD subjects with negative αSyn-SAA results by at least one of the groups, at least at one time point. We examined clinical and biomarker data for these negative PD subjects (Additional file 1: Table S2). One subject (#3020) was unanimously detected as negative αSyn-SAA by all three groups, and demonstrated rapid motor progression (UPDRS Part 3 increased by + 25 from BL to Y3), elevated SCOPA-AUT score of 17, and elevated baseline CSF NfL (166.4 pg/ml), suggesting possible MSA or alternative diagnosis to PD.

There were 4 HC subject samples with positive αSyn-SAA results by at least one group at either time point (Additional file 1: Table S3). For instance, AbbVie, Amprion, and the Caughey lab each detected a different HC as positive at a single time point. All three assays detected HC #3264 as positive (AbbVie at Y3, Amprion at BL and Y3, and Caughey at BL). We reviewed available clinical data for these healthy control subjects for signs of potential neurologic disease or other confounds. HC #3264 had possible rapid eye movement (REM)-sleep behavior disorder (RBD) based on RBD questionnaire score of 5 (with question 6 also positive) and an elevated SCOPA-AUT score of 8. This is a possible synucleinopathy prodromal state in this subject, which could explain the highly concordant positive result, but this could not be verified. There were no notable clinical features in the other positive HC subjects (Additional file 1: Table S3).

### SWEDD results

Baseline CSF samples from 20 SWEDD subjects were tested by all three laboratories. AbbVie αSyn-SAA resulted in 3/20 positive (15%), Amprion 4/20 (20%, plus 1 inconclusive result), and Caughey with 4/20 (20%). There were 2 SWEDD subjects that were detected as positive by all three groups (Additional file 1: Table S4). Both had negative DAT-SPECT imaging at baseline and therefore were initially classified as SWEDD. However, subsequent DAT imaging (after > 3 years) showed clear deficit of radioligand uptake, consistent with dopamine denervation and PD [10]. The diagnostic status of these subjects was changed to PD post αSyn-SAA analysis. Only one other SWEDD subject was detected as positive by all three groups (#3082); this subject may have RBD based on questionnaire.

### Comparison of assay kinetic parameters

**Fig. 3:**
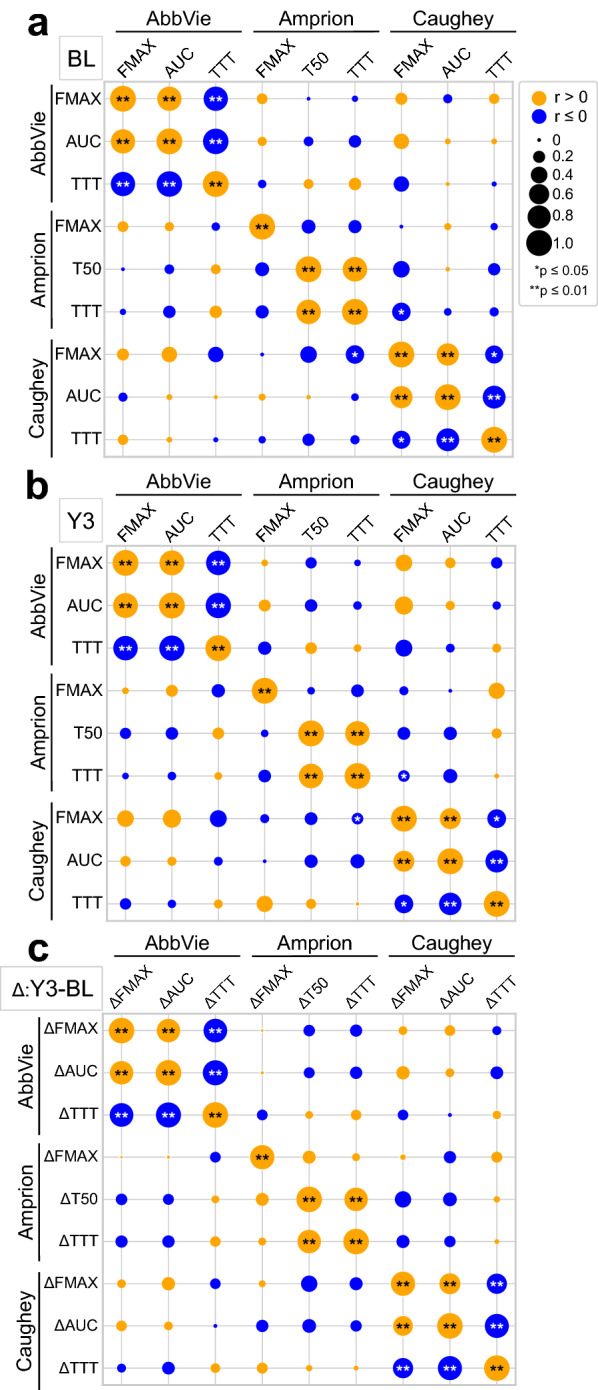
Correlations among αSyn-SAA kinetic parameters across the three assays. **a** Correlations among maximum fluorescence (*F*_max_), area under the curve (AUC), time to 50% maximum (T_50_), or time to threshold (TTT) among the three assays for baseline (BL) samples. Diameter of each circle is proportional to strength of correlation (Spearman r), and the color indicates positive (orange) and negative (blue) correlations (**p ≤ 0.01, *p ≤ 0.05). **b** Similar analysis of correlation for year 3 (Y3) samples. **c** Correlations of changes in assay kinetic parameters from baseline (BL) to year 3 (Y3). Absolute change (Δ, Y3—BL) was calculated and rank order correlations were determined for each pairing of parameters

αSyn-SAA produces a time-evolving fluorescence signal with a characteristic sigmoid shape as aggregation occurs. There is a well-defined lag-phase, exponential phase, and plateau phase. Several kinetic parameters are derived from this time-series data, including *F*_max_, AUC, TTT, and T_50_. Two of these measures, *F*_max_ and TTT were available for all three groups. We examined the variability of these parameters for PD patients with positive αSyn-SAA results. For each lab, the mean and range of *F*_max_ or TTT of replicates for each sample were determined (Additional file 1: Fig. S2), and we see high variability in *F*_max_ or TTT across technical replicates within a given assay. Furthermore, there are differences across the three assays, indicating significant variability for identical samples/patients (Additional file 1: Fig. S2). We explored whether kinetic parameters correlated across the three laboratories, which could imply that they are influenced by the seeding αSyn or the sample milieu. Using kinetic parameters derived from αSyn-SAA-positive PD samples, we evaluated for correlations among *F*_max_, AUC, T_50_, or TTT both within and among the three lab assays (Fig. 3). There were notable correlations among kinetic parameters within assays. For instance, the onset of the reaction (T_50_ or TTT) correlates inversely with *F*_max_—aggregation producing higher *F*_max_ was achieved in reactions that had more rapid onset (shorter lag phase). This has been observed for αSyn and earlier PrP assays, and is influenced by seed concentration, by other molecules [31], or by incomplete recording of curves with a long lag phase. Caughey αSyn-SAA *F*_max_ had modest inverse correlation to Amprion TTT. We did not otherwise observe significant correlations of kinetic parameters across the three assays. There were also no significant correlations when comparing the absolute change (Y3–BL) in assay kinetic parameters from BL to Y3 (Fig. 3c).

### Comparison of assay kinetics with clinical and other biomarker data

A secondary question is whether these assays can provide information about the severity of disease, clinical phenotype, or even prognosis. We utilized the available PPMI clinical and biomarker data to explore correlations to assay kinetic parameters across all three sets of results. Using data from PD subjects with positive αSyn-SAA, we calculated correlation coefficients of kinetic parameters (*F*_max_, AUC, T_50_, TTT) versus multiple clinical motor (UPDRS), non-motor (RBDQ, SCOPA-AUT, MoCA, UPSIT), imaging (DAT-SPECT SBR), and biomarkers (CSF Aβ, tau, and NfL) (Additional file 1: Fig. S3, online resource). There were some notable correlations between assay parameters and clinical metrics. For instance, disease duration positively correlated to Caughey αSyn-SAA *F*_max_ (*r* =  + 0.53, *p* = 0.007, *n* = 24), and negatively to Caughey TTT (*r* = − 0.46, *p* = 0.025, *n* = 24), and AbbVie αSyn-SAA TTT (*r* = − 0.42, *p* = 0.039, *n* = 25). The only significant correlations to motor scores were between Caughey BL *F*_max_ and BL AUC and Y3 UPDRS Part 3 Off Score (*r* =  + 0.51, *p* = 0.025 and *r* =  + 0.51, *p* = 0.025, respectively; *n* = 19). Interestingly, there was strong negative correlation between Amprion αSyn-SAA *F*_max_ and contralateral putamen SBR (*r* = − 0.64, *p* = 0.0006, *n* = 27) at baseline. Among the additional CSF biomarker data available, there were no consensus associations across the assays. There was positive correlation of CSF total αSyn to Amprion αSyn-SAA TTT (*r* =  + 0.68, *p* = 0.0005, *n* = 27), but only at Y3. There were no consistent trends across assays, across time-points, or within categories of clinical/biomarker data (Additional file 1: Fig. S3).

### End-point dilution αSyn-SAA

As an alternative method to derive quantitative information, the Caughey lab performed end-point dilution of positive PD samples. End-point dilution provides an estimate of the seed concentration within a biosample, and has been previously reported for prions [30] and αSyn [13] in a variety of tissues. Samples were serially diluted twofold and αSyn-SAA performed, and the dilution producing 50% positive replicates in a series was estimated and used to calculate the concentration of 50% seeding units or ‘doses’ (SD50) in the original CSF. This provides a quantitative measure that should linearly correspond to the concentration of αSyn seeds. End-point dilution was achieved for 19 of the αSyn-SAA positive samples from the Caughey group. SD50 positively correlates to normalized *F*_max_ (*r* =  + 0.49, *p* = 0.033, *n* = 19), but not to other kinetic parameters (Additional file 1: Fig. S5). We evaluated correlations between SD50 and baseline clinical features, including age, disease duration, motor (UDPRS Part 3) scores, non-motor scores, and additional biomarkers (CSF αSyn and NfL). We observed positive correlation between SD50 and NfL (*r* = + 0.51, *p* = 0.05, Additional file 1: Fig. S4a), but age accounts for part of this relationship (multiple linear regression with age and SD50 as independent variables: β_age_ = 3.9, *p* = 0.0013 and β_SD50_ = 9.1, *p* = 0.057). One hypothesis is that SD50 might increase with duration or progression of disease, but we did not observe consistent pairwise differences in SD50 between BL and Y3 (Additional file 1: Fig. S4b). We also examined change in NfL with time from BL to Y3, as this biomarker is associated with longitudinal progression of disease in another de novo cohort [32, 33]. Similarly to SD50, there was no change of NfL from BL to Y3 (Additional file 1: Fig. S4c) in this PPMI subset. Moreover, we did not see a relationship between baseline SD50 and change in motor scores from BL to Y3, or between change in SD50 from BL to Y3 and change in UPDRS Part 3 or total scores (Additional file 1: Fig. S4d). These subjects have relatively early disease, and SD50 may correlate to a wider range of the disease from early to advanced stages. We therefore included end-point dilution data for subjects from the BioFIND study, which enrolled and collected CSF from PD subjects with more advanced disease (Additional file 1: Fig. S6). Here, SD50 has positive correlation to age (*r* =  + 0.36, *p* = 0.006) and disease duration (*r* =  + 0.31, *p* = 0.02), but not meaningful associations with other clinical measures.

## Discussion

αSyn-SAAs performed in parallel by three groups demonstrate high diagnostic performance for PD, with 86–96% sensitivity and 97–100% specificity at baseline. Despite differences in methods and laboratories, all three assays achieved diagnostic accuracy that could provide practical clinical benefit for detecting early αSyn disease. While there were slight differences in sensitivity and specificity calculated for each group, this does not likely represent any systematic advantage or shortcoming of any single assay, but is more likely attributable to reasonable variability within this small sample size. Beyond diagnostic performance against clinically validated subjects, there was a remarkable unanimous concordance across all three assays for subjects whose initial diagnostic designation had changed. This highlights the high negative predictive value of the assays, and illustrates practical scenarios where an early negative αSyn-SAA result might prompt the clinician or investigator to consider alternative diagnoses, even when clinical or imaging data do not necessarily deviate from classical PD.

Initial diagnostic designations for each PPMI cohort are based upon clinical and imaging assessments relatively early in the disease course, but with time and disease progression, the clinical diagnosis may change. Diagnoses changed for 2 subjects within the PD group, both of whom had negative αSyn-SAA at both baseline and year 3 determined by *all three assays*. One of these patients (#3212) had rapid progression and early demise. Postmortem pathology confirmed a diagnosis of multiple system atrophy (MSA), with oligodendroglia αSyn inclusions. Another patient (#3027) was determined to have diagnosis of atypical parkinsonism not consistent with idiopathic PD after independent review. These cases illustrate the ability of αSyn-SAA to distinguish early PD from other forms of parkinsonism.

The negative result observed for the confirmed MSA patient forces the question as to why a known synucleinopathy does not produce detectable aggregation. MSA is associated with αSyn forms known to aggregate and propagate in the CNS, though with overwhelming tendency to form oligodendroglial intracellular inclusions. One hypothesis is that the major differences between PD and MSA are due to structural strain differences in the essential αSyn forms underlying each disease [34–36]. We do know that MSA-derived CSF behaves differently than PD-derived CSF in the αSyn-SAA. Depending on assay protocol, MSA-derived samples either do not show aggregation [24] or aggregation occurs with kinetic profile distinct from that of PD [26]. Aggregation of MSA samples begins earlier (lower T_50_ or TTT), and achieves a lower steady-state fluorescence (*F*_max_) than for PD. Moreover, this has only been observed under a specific set of reaction conditions [26]. This MSA sample was detected as negative in all three of these assays, which had been optimized for generating and detecting a typical PD profile, consistent with a previous study that also noted negative αSyn-SAA in 29 of 31 MSA cases [24]. With further optimization and validation, these assays have potential to distinguish MSA from PD [24] as well as controls [26].

There were 7 other PD subjects with αSyn-SAA results discordant from diagnosis and one of these (#3020) by all three labs, which could be considered false negatives. There were also 4 HCs that were variously detected as positive αSyn-SAA, and one of these (#3264) was unanimously detected as positive. These are considered false positive results. When reviewing their clinical data, there was nothing to indicate neurologic abnormality, with exception of #3264 who might be considered to have REM-sleep-behavior disorder (RBD) with a RBD questionnaire score of 5 (with positive question #6). Out of 6 HC subjects with probable RBD based on the RBD questionnaire total (≥ 5) or positive response to dream-enactment (positive question #6) from the tested cohort, 2 (#3074 and #3264) showed positive αSyn-SAA (33%). Likewise, out of 12 HC subjects in the tested cohort with SCOPA-AUT score ≥ 7, 2 (17%) showed positive αSyn-SAA (#3112 in Amprion Y3, and #3264 by all groups). Several studies have now noted much higher positivity rates of αSyn-SAA in prodromal cases [24, 37, 38]. Up to 59% of patients with RBD confirmed by polysomnography can have DAT deficit at baseline [39], vs. none in the PPMI control subjects with RBD symptoms. The relatively lower rate of αSyn-SAA positivity in these control subjects with incidental RBD symptoms or dysautonomia is likely to be due to selection bias of published studies focused on prodromal cohort vs. those with incidentally noted symptoms, and the fact that we do not have objective autonomic testing or polysomnography (PSG) in these subjects for confirmed diagnosis, although questionnaires have been shown to have at least moderate correlation with objective testing results [40–43].

All three assays generated positive αSyn-SAA results for 2 SWEDD subjects with clinical features of parkinsonism, but borderline baseline DAT-SPECT imaging. Both subjects had later DAT-SPECT at 3 years that unequivocally demonstrated significant striatal denervation [10]. Again, this emphasizes the power of αSyn-SAA to detect PD early during the disease course, potentially even before substantia nigra degeneration occurs. These SWEDD subjects did have early motor signs of parkinsonism, indicating manifest nigrostriatal dysfunction, but the relative preservation of dopaminergic terminals by imaging suggests that diagnosis by αSyn-SAA may be possible before significant neuron loss. αSyn-SAA has already been tested in several cohorts of prodromal synucleinopathies, including RBD or pure autonomic failure, detecting seeding-competent αSyn forms before definite motor or cognitive phenotypes emerge [24, 37, 38, 44]. Decreased binding of radioligand can indicate disruption of the nigrostriatal pathway, but this is not specific to PD or to αSyn-dependent degenerative processes, as this can be seen in other forms of parkinsonism, such as PSP or CBD. It also cannot provide evidence of abnormalities of other brain areas or changes that precede nigrostriatal dysfunction. αSyn-SAAs can distinguish these subjects based on the principle that αSyn pathology is infixed before motor signs emerge and detect PD among SWEDD subjects.

A limitation of this study was lack of available autopsy data to provide a definitive pathologic diagnosis for validation of baseline αSyn-SAA results. Neuropathology was available for only two patients, one with MSA as described above, and another (#3076) who had mixed Lewy body and Alzheimer pathology and that was detected as αSyn-SAA-positive by all three groups. There have been several important studies by other groups of αSyn-SAA of CSF [6, 24, 45] or skin [12, 17, 45] of autopsy-confirmed patient cohorts, with comparable diagnostic performance. We had sought to validate SAAs within an early PD cohort, which has correspondingly limited neuropathologic data from autopsy.

### Quantitative information from αSyn-SAA

Kinetic parameters of each αSyn-SAA set were compared to clinical, imaging, and biomarker data available for each patient. With the development of prion SAAs to determine presence of prions seeds in prion disease, there is evidence that the kinetics of the aggregation reaction and evolution of the corresponding fluorescence signal is related to the number of seeds initially added to the reaction, or the aggregation propensity of the seeds [9, 13, 30]. Aggregation occurs earlier and produces higher maximum fluorescence with more initial seeds, resulting in shorter T_50_, shorter TTT or higher *F*_max_. We asked whether assay kinetic parameters, namely *F*_max_, T_50_, TTT, or AUC, correlated to any particular clinical features, with the broad assumption that these could indicate variations in seeding potential that would also correlate to clinical severity or other markers of disease progression. However, we observe substantial variability that likely limits the quantitative utility of αSyn-SAA for CSF as they are performed now. For instance, there is significant variability of fluorescence amplitude and time course in aggregation reactions run from the same sample (replicates). There is also significant variability across the three labs. This could be due to inherent variability of the aggregation process, differences in reagents or procedures, heterogeneous analyte related to the extremely low concentrations of the biomarkers, and relatively low numbers of samples analyzed. In addition, each laboratory has different reaction conditions, as summarized in Table 1.

We nonetheless examined possible relationships of αSyn-SAA parameters to multiple clinical measures, including UPDRS sub-scores and total score, UPSIT, RBD, SCOPA, and MoCA scores, DATSCAN specific binding ratios, and CSF αSyn and NfL. Despite extensive analyses of assay parameters from all three laboratories, across these multiple clinical variables, and across baseline and year 3 data, we cannot draw any substantial conclusions as to the quantitative value of assay kinetics. There were some potentially meaningful correlations that suggest a link to underlying severity, such as Amprion *F*_max_ inversely correlating to contralateral putamen signal, or Amprion and AbbVie *F*_max_ inversely correlating to UPSIT (*i.e.* more impaired olfaction correlates to higher *F*_max_). However, results were not consistent across all three assays.

The Caughey lab extended this quantitative analysis by performing end-point dilution on positive PD samples. SD50 may provide direct measure of the initial seed concentration or seeding potential of the biomatrix [13]. Here, we observed modest positive correlation (*r* =  + 0.51, *p* = 0.05, *n* = 16) between SD50 and NfL at baseline, which is a biologically plausible association between higher seeding activity and a biomarker known to faithfully indicate neural and particularly axonal injury, but this should be considered within the context of relationships of SD50 and NfL to age [46]. This could not be confirmed with the extended BioFIND data set, for which NfL measurements are unavailable. Apart from SD50 correlating to age and disease duration, we did not see additional clinical correlations among the pooled PPMI and BioFIND end-point dilutions.

There are multiple potential explanations for the difficulty in drawing robust clinical correlations from these assays. Seed amplification assays are inherently non-linear processes, and slight variations in the initial conditions, which include experimental factors, seed concentration, seed conformation, additional proteins or small molecules within the reaction milieu, can have large effects on the kinetics of the aggregation reaction or even prevent successful aggregation. Even under identical assay conditions, two identical samples may not precisely reproduce the aggregation kinetics due to variability between technical replicates. Unlike polymerase chain reaction (PCR), for instance, the recurrent cycles of aggregation and disruption do not obey a ratiometric and predictable templating, but are subject to significant variability in the fracturing and formation of new seeds and new seeding surfaces with each cycle. Considering these sources of intrinsic assay variability, it is remarkable that the binary diagnostic performance was so consistently high across all three laboratories.

The biology of the seeds may be also responsible for the lack of quantitative differences over time and across the severity of the diseases in PD cohorts. Emerging evidence that αSyn-SAA is positive in prodromal cases including in our few example cases may indicate that pathological seeds are present very early in the course of synucleinopathies and plateau by the time detectable symptoms emerge. Further studies are essential to explore quantitative assays in early prodromal stages. Another interesting and important question that has not yet been addressed is whether therapies targeted to αSyn change the αSyn-SAA assay results.

It remains undetermined whether assay kinetic parameters or end-point dilution data can provide information about disease severity, clinical features, or clinical subtypes, but our results suggest that the quantitation will require additional statistical power and careful normalization. There have been efforts to standardize the assay, and one approach relies on stabilized ultra-short fibrils to act as a reference standard of known concentration and kinetics to guide interpretation of biologic samples [47]. We provide preliminary data that there may be clinical information to glean from αSyn-SAA beyond the diagnostic result. An obvious limitation of this correlation analysis is the low sample size. Quantitation may only be possible from multivariate analysis of a much larger data set of PD patients.

The diagnostic value of seed amplification assays for PD and related synucleinopathies has now been demonstrated in many independent studies of PD subjects. We have established that the assay is robust to methodological differences when performed by experienced hands, validating the diagnostic interpretations of the assay and further illustrating the maturation of αSyn-SAA as a clinical diagnostic tool even in early de novo PD when diagnosis is often more difficult. αSyn-SAA will augment current clinical diagnosis, provide important adjunctive support to equivocal cases of parkinsonism beyond what is possible with current imaging, and promises to provide early etiologic identification of patients who may most benefit from αSyn-targeted neuroprotective strategies.

## Supplementary Information

**Additional file 1. Tables S1-S4 and Figures S1-S6.**

## Data Availability

Assay data from all three groups are available in the PPMI LONI database: https://www.ppmi-info.org/access-data-specimens/download-data (AbbVie: project #173; Amprion: project #155; Caughey: project #172).
