# Correction to: High diagnostic performance of independent alpha-synuclein seed amplification assays for detection of early Parkinson’s disease

**DOI:** 10.1186/s40478-021-01292-6

**Published:** 2021-11-26

**Authors:** Marco J. Russo, Christina D. Orru, Luis Concha-Marambio, Simone Giaisi, Bradley R. Groveman, Carly M. Farris, Bret Holguin, Andrew G. Hughson, David-Erick LaFontant, Chelsea Caspell-Garcia, Christopher S. Cofey, Jennifer Mollon, Samantha J. Hutten, Kalpana Merchant, Roland G. Heym, Claudio Soto, Byron Caughey, Un Jung Kang

**Affiliations:** 1 The Marlene and Paolo Fresco Institute for Parkinson’s & Movement Disorders, Department of Neurology, Department of Neuroscience and Physiology, Neuroscience Institute, The Parekh Center for Interdisciplinary Neurology, NYU Grossman School of Medicine, New York, NY USA; 2 Laboratory of Persistent Viral Diseases, Rocky Mountain Laboratories, National Institute of Allergy and Infectious Diseases, National Institutes of Health, Hamilton, MT USA; 3 R&D Unit, Amprion Inc., San Diego, CA USA; 4 AbbVie Deutschland GmbH & Co. KG, Ludwigshafen, Germany; 5 Department of Biostatistics, College of Public Health, University of Iowa, Iowa City, IA USA; 6 Michael J. Fox Foundation for Parkinson’s Research, New York, NY USA; 7 Northwestern University Feinberg School of Medicine, Chicago, IL USA; 8 Mitchell Center for Alzheimer’s Disease and Related Brain Disorders, Department of Neurology, University of Texas Houston Medical School, Houston, TX USA

## Correction to: acta neuropathol commun (2021) 9:179 https://doi.org/10.1186/s40478-021-01282-8

During the publication process of the original article [1] an error was made and several corresponding authors were not shown.

The incorrect corresponding author was:

- Un Jung Kang

The correct corresponding authors are:

- Luis Concha-Marambio, Roland G. Heym, Claudio Soto, Byron Caughey and Un Jung Kang

The publisher apologizes for the inconvenience caused to the authors and readers.
