# Supplementary material for: High diagnostic performance of independent alpha-synuclein seed amplification assays for detection of early Parkinson’s disease
Source: Acta Neuropathol Commun. 2021 Nov 6;9:179. doi: 10.1186/s40478-021-01282-8 (PMC8572469; doi:10.1186/s40478-021-01282-8)
Supplement: Supplementary file 1 — Additional file 1. Tables S1-S4 and Figures S1-S6. [file 40478_2021_1282_MOESM1_ESM.pdf]

High diagnostic performance of independent alpha-synuclein seed amplification assays for detection of early Parkinson's disease

Marco J. Russo[1]*, Christina D. Orru[2]*, Luis Concha-Marambio[3*¥], Simone Giaisi[4]*, Bradley R. Groveman[2], Carly M. Farris[3], Bret Holguin[3], Andrew G. Hughson[2], David-Erick LaFontant[5], Chelsea Caspell-Garcia[5], Christopher S. Coffey[5], Jennifer Mollon[4], Samantha J. Hutten[6], Kalpana Merchant[7], Roland G. Heym[4¥], Claudio Soto[3,8¥], Byron Caughey[2¥], Un Jung Kang[1¥].
*Authors contributed equally.

Supplementary Information

Supplementary Tables 1-4

Table S1. Demographic and clinical data for randomly selected cohorts.

Table S2. Clinical features of PD subjects with negative/inconclusive αSyn-SAA results.

Table S3. Clinical features of HC subjects with positive αSyn-SAA results.

Table S4. Clinical features of SWEDD subjects with positive αSyn-SAA results.

Supplementary Figures 1-6

Figure S1. Raw numbers of positive, negative, or inconclusive αSyn-SAA results by three laboratories. Figure S2. Variability of αSyn-SAA fluorescence kinetic parameters.

Figure S3. Correlations of SAA kinetic parameters to clinical, imaging, and biomarker data.

Figure S4. End-point dilution αSyn-SAA comparison to clinical data.

Figure S5. End-point dilution SD50 relationship to other αSyn-SAA kinetic parameters.

Figure S6. SD50 correlations to clinical data for PPMI and BioFIND subjects.

Table S1

	PD			Healthy Controls			SWEDD		
	SAA Cohort	PPMI	*p*	SAA Cohort	PPMI	*p*	SAA Cohort	PPMI	*p*
**n**	28	421		30	196		18	62	
Age (years)	62.1 (9.3)	61.7 (9.7)	*0.803*	63.8 (10.6)	60.8 (11.2)	*0.167*	59.6 (10.6)	61.3 (10.0)	*0.519*
Gender (M/F)	19 / 9	275 / 146	*0.798*	18 / 12	126 / 70	*0.651*	12 / 6	39 / 23	*0.823*
Duration (months)	9.0 (8.4)	6.7 (6.5)	*0.075*	-	-	-	8.1 (6.4)	6.8 (7.2)	*0.392*
Age (onset)	60.0 (9.2)	59.7 (10.0)	*0.843*	-	-	-	57.1 (10.6)	59.2 (10.5)	*0.418*
Age (diagnosis)	61.4 (9.1)	61.1 (9.7)	*0.882*	-	-	-	59.0 (11.0)	60.7 (10.2)	*0.499*
H&Y Stage	1.6 (0.5)	1.6 (0.5)	*0.444*	-	-	-	1.3 (0.5)	1.5 (0.5)	*0.143*
UPDRS Part 3	20.5 (8.6)	20.9 ± 8.9	*0.821*	2.6 (3.6)	1.2 (2.2)	*0.003*	12.1 (9.4)	14.4 (9.9)	*0.311*
UPDRS Total	33.9 (13.9)	32.4 (13.1)	*0.559*	6.5 (5.5)	4.6 (4.4)	*0.031*	28.1 (16.1)	28.5 (17.5)	*0.936*
UPSIT Score	22.5 (8.1)	22.4 (8.2)	*0.927*	35.4 (3.7)	34.0 (4.9)	*0.126*	32.7 (7.2)	31.3 (6.3)	*0.450*
RBD Score	4.8 (2.6)	4.1 (2.7)	*0.206*	3.1 (1.9)	2.8 (2.3)	*0.581*	3.7 (2.2)	4.6 (2.9)	*0.108*
SCOPA-AUT	11.0 (6.7)	9.5 (6.2)	*0.232*	6.3 (4.1)	5.8 (3.7)	*0.523*	14.5 (7.8)	14.0 (8.9)	*0.796*
MoCA Score	27.0 (2.2)	27.1 (2.3)	*0.717*	27.9 (1.0)	28.2 (1.1)	*0.169*	27.9 (1.7)	27.0 (2.4)	*0.886*
DAT SBR Mean Caudate	1.9 (0.6)	2.0 (0.6)	*0.432*	2.8 (0.6)	3.0 (0.6)	*0.171*	3.1 (0.6)	2.9 (0.6)	*0.259*
DAT SBR Mean Putamen	0.7 (0.2)	0.8 (0.3)	*0.141*	1.9 (0.5)	2.1 (0.6)	*0.211*	2.2 (0.5)	2.1 (0.5)	*0.327*
CSF Aβ (pg/ml)	854.4 (356.2)	909.6 (410.7)	*0.489*	926.6 (443.1)	1019.4 (499.5)	*0.399*	990.4 (382.4)	952.9 (354.4)	*0.208*
CSF αSyn (pg/ml)	1449.1 (723.2)	1506.7 (666.6)	*0.66*	1709.9 (788.4)	1695.2 (747.4)	*0.921*	1652.9 (830.1)	1678.8 (725.1)	*0.651*
CSF tau (pg/ml)	179.0 (61.8)	169.5 (56.8)	*0.411*	204.2 (104.1)	191.6 (79.3)	*0.441*	174.8 (62.0)	179.8 (60.0)	*0.678*
CSF p-tau (pg/ml)	15.9 (6.6)	14.9 (5.2)	*0.364*	19.5 (12.8)	17.5 (8.3)	*0.281*	15.2 (5.2)	15.7 (6.0)	*0.686*
CSF NfL (pg/ml)	101.1 (45.0)	102.3 (57.2)	*0.922*	118.4 (50.2)	98.7 (55.1)	*0.133*	-	-	-

Table S1. Demographic, clinical, imaging, and biomarker data were reviewed for the cohorts tested in this study, which were randomly selected from the larger PPMI study population. Data for each cohort are presented as mean (SD) alongside the parent population from which they were selected. We did not observe statistically significant differences between the smaller subset and the remaining population (p value for 2-sided t-test is shown), with exception of small difference for UPDRS Part 3 healthy controls (3.1 ± 1.9 vs. 2.8 ± 2.3). Note that the 2 PD and 2 SWEDD subjects who had diagnoses changed during the study were removed from this table. There were no significant differences between the HC and PD tested cohorts for age ($p = 0.517$) or gender ($p = 0.542$). PD and SWEDD tested cohorts were balanced for age ($p = 0.317$), gender ($p = 0.775$), duration ($p = 0.775$), age of onset ($p = 0.238$), or age of diagnosis ($p = 0.334$).

Table S2

	3018			3020			3086			3119			3134			4103			3666			3027			3212		
Diagnosis	PD			PD			PD			PD			PD			PD			PD			PD → Not PD			PD →MSA		
	Abb	Amp	Cau	Abb	Amp	Cau	Abb	Amp	Cau	Abb	Amp	Cau	Abb	Amp	Cau	Abb	Amp	Cau	Abb	Amp	Cau	Abb	Amp	Cau	Abb	Amp	Cau
SAA Result BL	⊕	⊕	⊖	⊖	⊖	⊖	⊖	∅	⊕	⊕	∅	⊕	⊕	⊕	⊕	⊖	⊕	⊖	⊕	⊕	⊖	⊖	⊖	⊖	⊖	⊖	⊖
SAA Result Y3	⊕	⊕	⊕	⊖	⊖	⊖	⊕	⊕	⊕	⊕	⊕	⊕	⊕	⊕	⊖	⊖	⊕	⊕	⊕	⊕	⊖	⊖	⊖	⊖	⊖	⊖	⊖

	3018		3020		3086		3119		3134		4103		3666		3027		3212	
DaTScan	Positive		Positive		Positive		Positive		Positive		Positive		Positive		Positive		Positive	
	ipsi	contra	ipsi	contra	ipsi	contra	ipsi	contra	ipsi	contra	ipsi	contra	ipsi	contra	ipsi	contra	ipsi	contra
Caudate SBR	1.96	1.58	1.92	1.54	2.52	2.21	1.84	1.75	1.93	1.42	2.30	2.3	1.63	1.39	2.88	2.74	1.7	1.41
Putamen SBR	0.60	0.37	0.83	0.82	1.22	0.88	0.51	0.37	1.06	0.66	1.17	0.67	0.79	0.51	1.88	1.28	0.73	0.42

	3018	3020	3086	3119	3134	4103	3666	3027	3212
Age/Gender	61 M	74 M	56 M	64 F	39 F	59 F	52 F	70 F	56 F
Onset Age	60	70	54	62	38	58	45	68	55
UPDRS Part 3 (off) BL	19	20	7	25	8	7	15	23	18
UPDRS Part 3 (off) Y3	38	45	13	33	29	18	24	26	52
UPDRS Part 3 (off) Δ	+19	+25	+6	+8	+21	+11	+9	+3	+34
UPDRS Total (off) BL	31	45	15	38	12	12	52	40	39
UPDRS Total (off) Y3	57	83	34	48	35	37	45	48	87
UPDRS Total (off) Δ	+26	+38	+19	+10	+23	+25	-7	+8	+48
RBD	Negative (3) Q6+	Positive (6) Q6+	Positive (5) Q6+	Negative (4) Q6+	Positive (8) Q6+	Positive (5) Q6-	Positive (5) Q6+	Positive (7) Q6+	Positive (10) Q6+
SCOPA-AUT	8	17	10	6	5	3	15	16	15
UPSIT	26 Hyposmia	26 Hyposmia	15 Anosmia	30 Hyposmia	31 Hyposmia	32 Hyposmia	38 Normosmia	30 Hyposmia	35 Normosmia
MoCA	23	28	24	28	29	27	29	25	23
CSF αSyn [pg/ml] BL	1379.8	2348.6	1156.4	1220.8	2028.2	2030.4	1509.5	1903.4	978.3
CSF αSyn [pg/ml] Y3	na	1977.8	na	1368.1	na	1813.2	na	na	916.3
CSF NfL [pg/ml] BL	na	166.4	71.92	48.42	na	83.62	89.99	159.4	113.4
CSF NfL [pg/ml] Y3	na	na	na	44.26	na	97.63	na	na	277.0

Table S2. Clinical features of PD subjects with negative/inconclusive αSyn-SAA results by at least one assay. Data for all PD diagnoses during this study (and unanimously negative SAA) are also presented in rightmost columns (#3027 and #3212)

Table S3

		3053			3074			3112			3264		
Group		HC			HC			HC			HC		
		Abb	Amp	Cau	Abb	Amp	Cau	Abb	Amp	Cau	Abb	Amp	Cau
SAA Result	BL	⊖	⊖	⊖	⊖	⊖	⊖	⊖	⊖	⊖	⊖	⊕	⊕
	Y3	⊖	⊖	⊕	⊕	⊖	⊖	⊖	⊕	⊖	⊕	⊕	⊖

		3053		3074		3112		3264	
DaTScan		Negative		Negative		Negative		Negative	
		R	L	R	L	R	L	R	L
Caudate SBR		2.11	2.26	2.57	2.54	2.48	2.76	3.25	3.02
Putamen SBR		1.15	1.58	1.38	2.02	1.77	1.56	2.5	2.34
Age/Gender		69 F		31 F		63 M		60 M	
UPDRS Part 3	BL	3		1		0		0	
	Y3	3		0		0		3	
	Δ	0		-1		0		+3	
UPDRS Total	BL	4		3		0		7	
	Y3	4		0		0		5	
	Δ	0		-3		0		-2	
RBD		Negative (2) Q6-		Negative (4) Q6+		Negative (0) Q6-		Positive (5) Q6+	
SCOPA-AUT		3		1		7		8	
UPSIT		34 Hyposmia		39 Normosmia		37 Normosmia		37 Normosmia	
MoCA		29		28		30		29	
CSF αSyn [pg/ml]		1381.4		844.6		1522		1702.6	
CSF NfL [pg/ml]		172.1		23.07		na		na	

Table S3. Healthy controls with positive αSyn-SAA results. Clinical features of healthy control subjects who had positive SAA results by at least one assay and one time point. Three of these (#3053, #3074, and #3112) had only single positive SAA result. Another subject was positive for RBD with SCOPA-AUT > 7, suggesting possible prodromal state.

Table S4

	3082			3256			3319			3384			3050			3101		
Group	SWEDD			SWEDD			SWEDD			SWEDD			SWEDD→PD			SWEDD→PD		
	Abb	Amp	Cau	Abb	Amp	Cau	Abb	Amp	Cau	Abb	Amp	Cau	Abb	Amp	Cau	Abb	Amp	Cau
SAA Result	⊕	⊕	⊕	⊖	⊖	⊕	⊖	∅	⊖	⊖	⊕	⊖	⊕	⊕	⊕	⊕	⊕	⊕

	3082		3256		3319		3384		3050		3101	
DaTScan	Negative		Negative		Negative		Negative		Neg → Pos		Neg → Pos	
	ipsi	contra	ipsi	contra	ipsi	contra	ipsi	contra	ipsi	contra	ipsi	contra
Caudate SBR	2.39	2.11	3.83	3.60	3.75	3.51	3.72	4.12	2.13	1.82	2.42	2.00
Putamen SBR	1.21	1.79	2.56	2.51	3.08	2.53	2.46	3.24	1.75	1.04	1.43	1.02
Age/Gender	66 M		57 M		53 F		66 F		52 M		50 F	
Onset Age	61		53		53		66		49		46	
UPDRS Part 3 (off)	4		24		13		11		18		5	
UPDRS Total (off)	16		38		28		32		29		12	
RBD	Positive (5) Q6+		Negative (3) Q6-		Negative (3) Q6+		Negative (2) Q6-		Negative(4) Q6-		Negative (3) Q6-	
SCOPA-AUT	14		21		9		18		6		6	
UPSIT	29 Hyposmia		33 Hyposmia		38 Normosmia		36 Normosmia		34 Hyposmia		31 Hyposmia	
MoCA	28		24		29		28		30		30	
CSF αSyn [pg/ml]	2140.3		4041.4		1387.9		2560		1354.4		1370.2	

Table S4. SWEDDs with positive αSyn-SAA results. Clinical data for SWEDD subjects with at least one positive/inconclusive SAA result. The two SWEDD subjects with diagnoses revised to PD on the basis of later DAT-SPECT imaging are shown at right (#3050 and #3101).

Figure S1

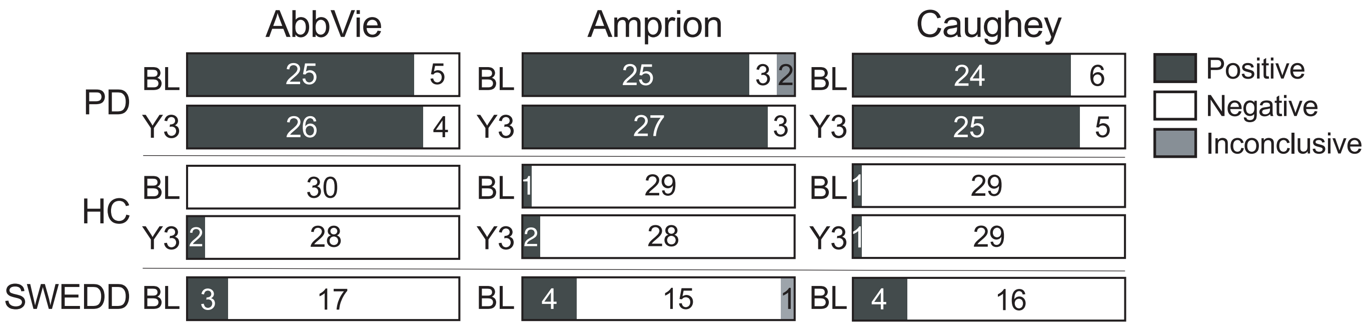

Fig. S1 Raw numbers of positive, negative, or inconclusive αSyn-SAA results by all three laboratories for Parkinson's disease (PD), healthy controls (HC), and subjects with scans without evidence of dopaminergic deficit (SWEDD), at baseline (BL) and year 3 (Y3). Note that these numbers include PD and SWEDD subjects with revised diagnoses that were removed from further analysis.

Figure S2

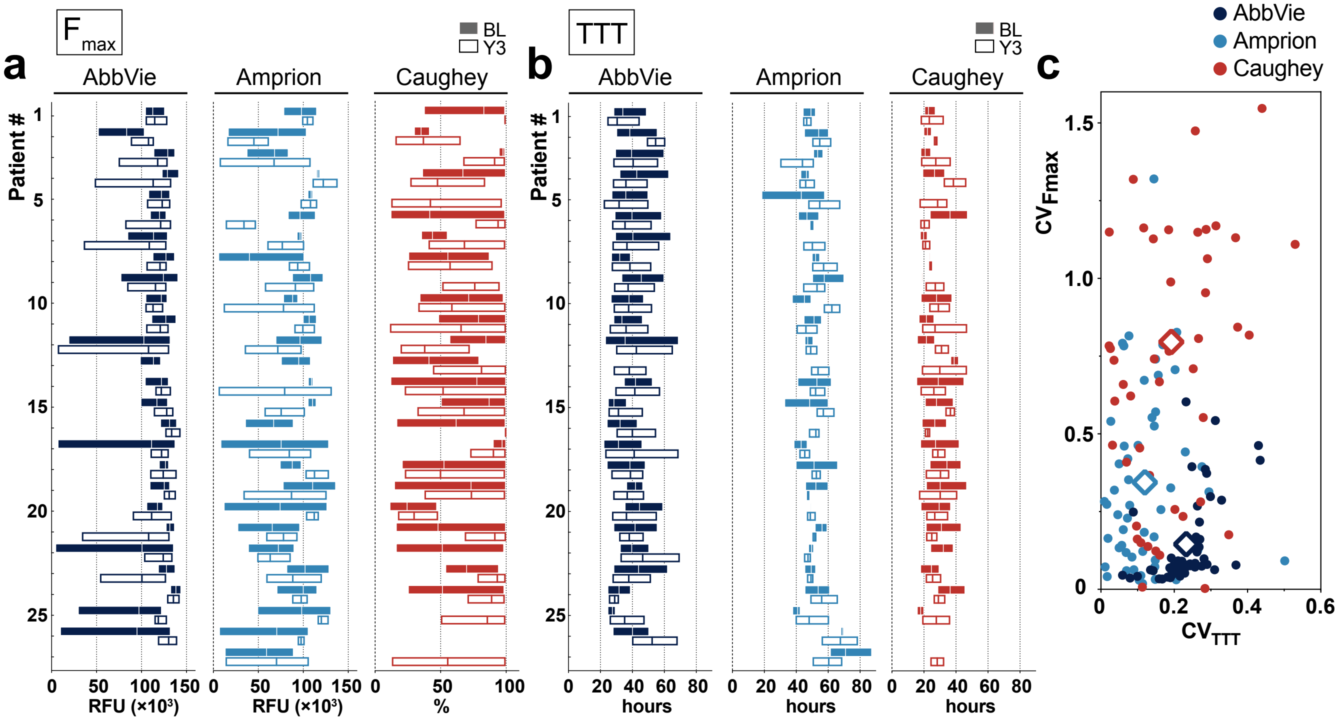

Fig. S2 Variability of αSyn-SAA fluorescence kinetic parameters. **a** Mean, maximum, and minimum of F_{max} obtained for each patient sample, with both BL (filled boxes) and Y3 (open boxes), for each assay. Note that Caughey lab normalized F_{max} to the maximal fluorescence on each plate, so this is expressed as a percentage of maximum. **b** Mean, maximum, and minimum of time to threshold (TTT) for each patient sample, for each assay. **c** Summary of variability, with CV_{Fmax} plotted against CV_{TTT} for AbbVie, Amprion, and Caughey, with each point representing a different sample (BL and Y3 pooled), and with the centroid for each represented by a diamond (CV_{Fmax}/CV_{TTT}, AbbVie: 0.15/0.23; Amprion: 0.34/0.12; Caughey: 0.80/0.19). Note the difference in xy scales.

Figure S3

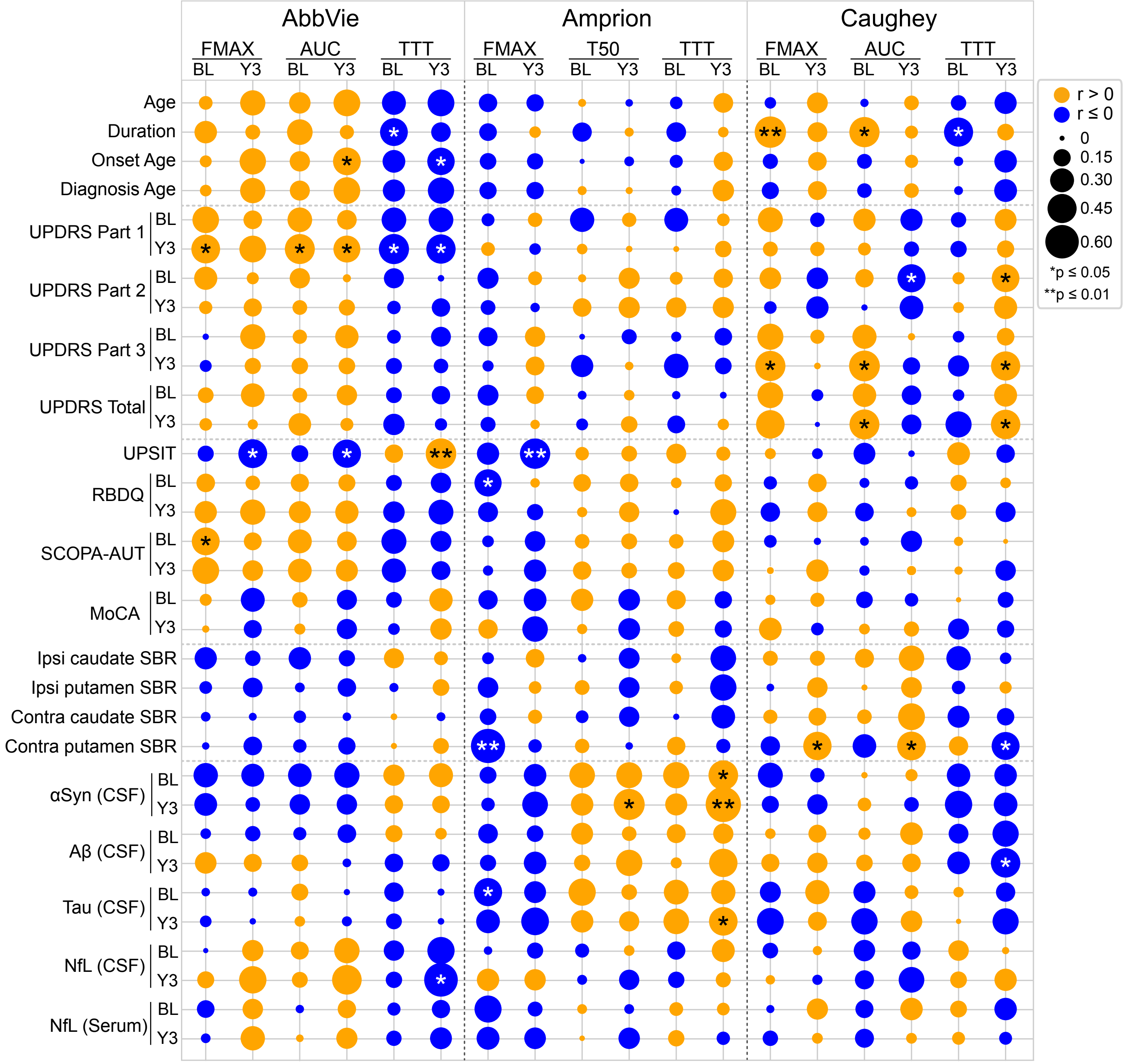

Fig. S3 Correlations of αSyn-SAA kinetic parameters to clinical, imaging, and biomarker data. Correlations of maximum fluorescence (F_{max}), area under the curve (AUC), time to 50% F_{max} (T_{50}), and time to threshold (TTT) from all three laboratories to clinical data (age, disease duration, age of onset/diagnosis, UPDRS sub-scores and total, UPSIT, MoCA, SCOPA-AUT, and RBDQ scores), imaging data (DATSCAN specific binding ratio), and biomarkers (Aβ, tau, total αSyn, and NfL). Diameter of circle at each node is proportional to strength of correlation (r), and the color indicates positive (orange) and negative (blue) correlations (**p ≤ 0.01, *p ≤ 0.05).

Figure S4

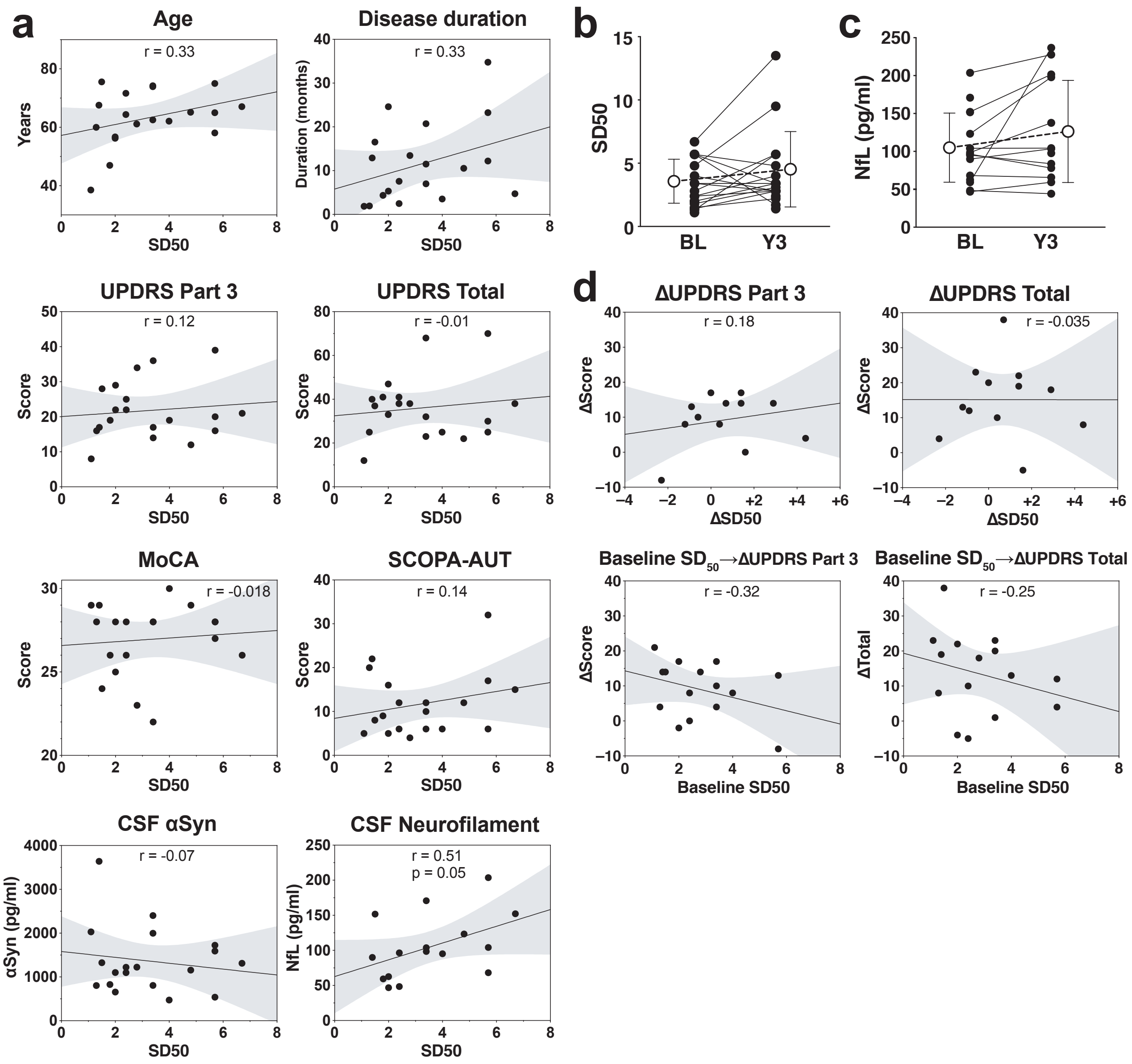

Fig. S4 End-point dilution SAA comparison to clinical data. **a** End-point dilution achieved for 19 PD samples. Relative concentration of SAA seeds (SD50/15 µl CSF) is plotted against the age, disease duration (months), UPDRS Part 3 ('off') and total scores, MoCA, SCOPA-AUT, CSF αSyn and CSF neurofilament light chain (NfL). Correlation coefficients (Spearman r) are provided for each plot, and p-value is indicated only for p ≤ 0.05). **b** SD50 values at baseline and year 3 (BL: 3.2 ± 1.7, n=19, Y3: 4.2 ± 3.0, n=18, mean ± s.d.; n.s., p = 0.13, Wilcoxon signed rank test). **c** Baseline and year 3 values for CSF neurofilament light chain, a biomarker that has been shown to correlate to disease progression and severity in other cohorts (BL: 104.7 ± 45.5, n=16; Y3: 126.0 ± 67.2, n=13, mean ± s.d., n.s., p=0.08, Wilcoxon signed rank test). **d** SD50 concentration (per 15 µl) vs. change in motor scores (UPDRS Part 3 and Total). ΔSD50 is plotted against ΔUPDRS Part 3 or Total, and baseline SD50 concentration is plotted against Δ UPDRS Part 3 or Total with correlation coefficient indicated for each plot.

Figure S5

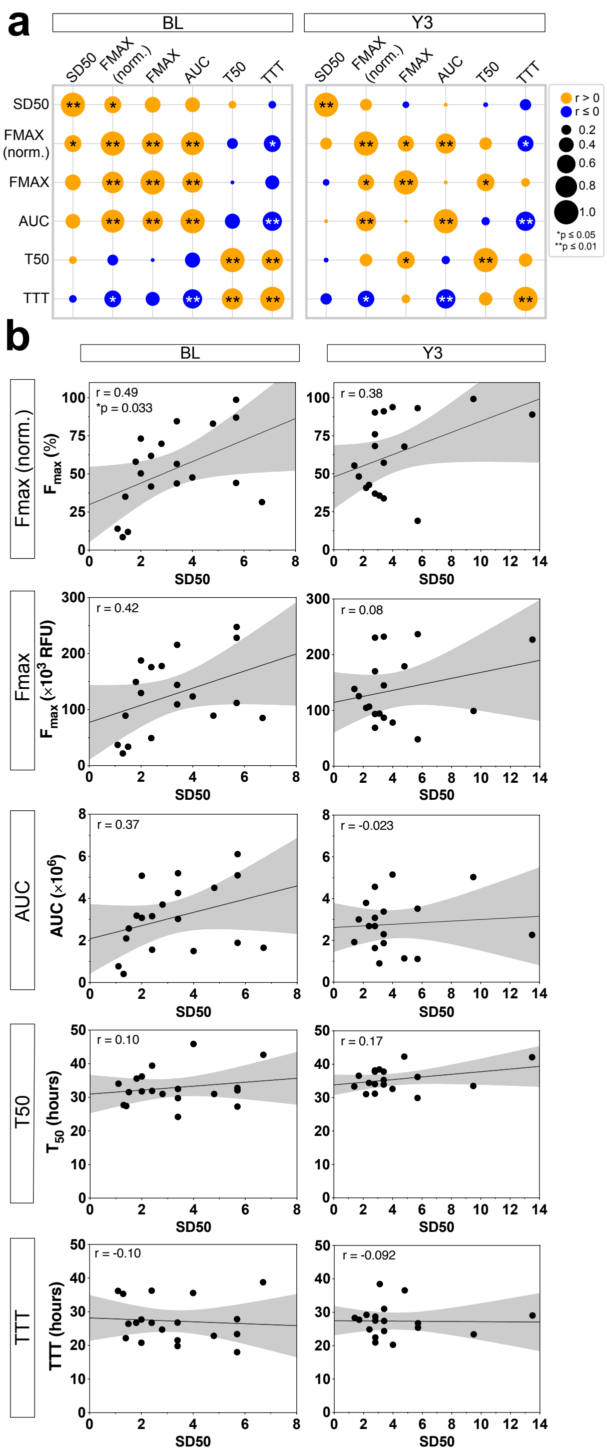

Fig. S5 End-point dilution SD50 relationship to other αSyn-SAA kinetic parameters. **a** Summary of SD50 vs. other αSyn-SAA kinetic parameters at baseline (BL) and year 3 (Y3), with each circle representing Spearman rank correlation and diameter indicating correlation magnitude (orange positive coefficient, and blue negative). **b** Scatter plots of Fmax, AUC, T50, and TTT vs. SD50 at both BL (left) and Y3 (right). Fmax (norm.) is fluorescence normalized to peak maximal fluorescence on plate. Linear best-fit and 95% confidence bands are shown for each pair. Spearman rank coefficient r is shown for each pair, and p value is shown if significant at p ≤ 0.05.

Figure S6

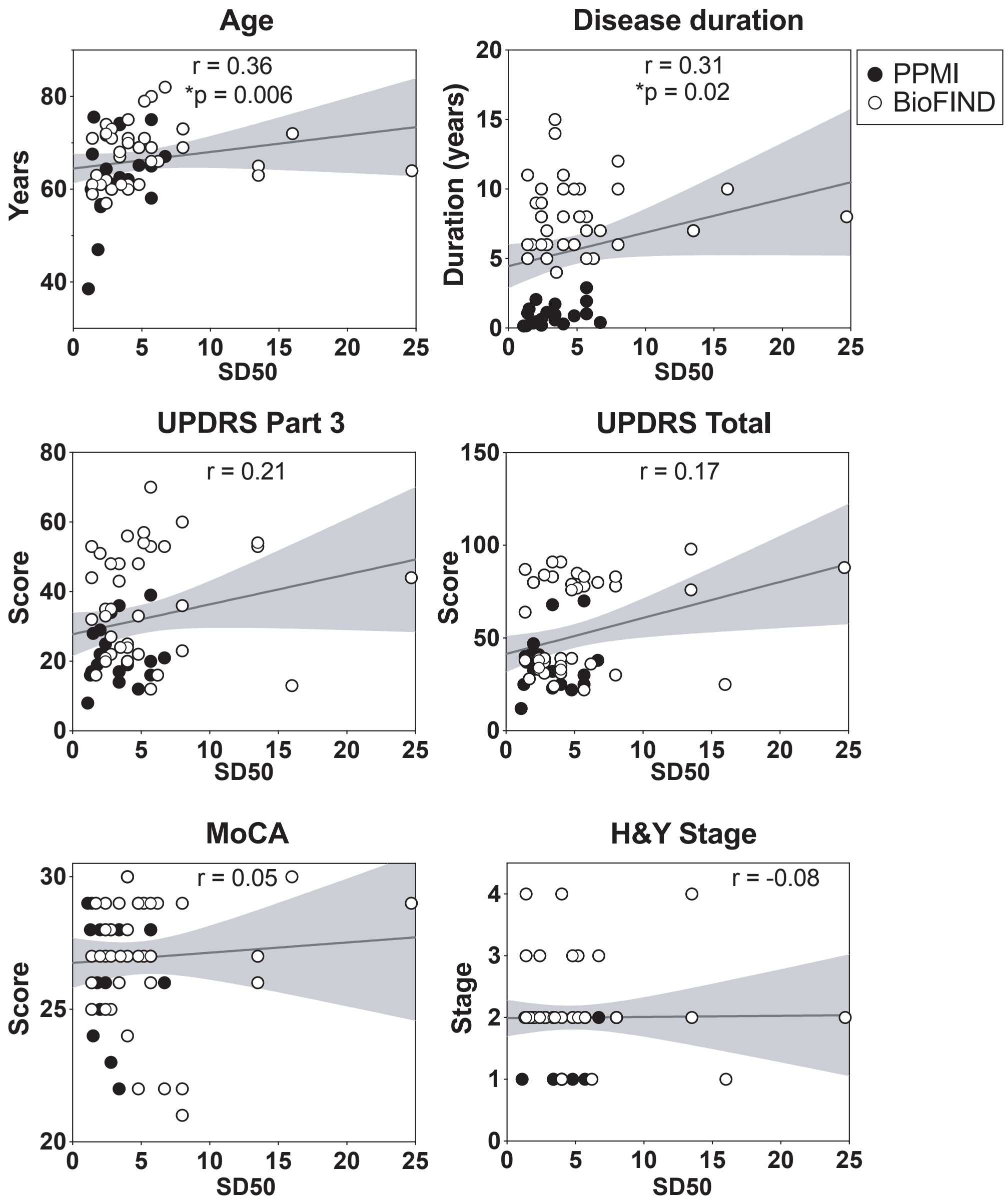

Fig. S6 SD50 correlations to clinical data for PPMI and BioFIND subjects. End-point dilution was performed on CSF from the BioFIND study, which includes PD subjects with more advanced disease. We asked whether these subjects with overall more severe clinical features, would provide additional power to detect correlations between SD50 and clinical parameters. With the exception of age (r = 0.36, p = 0.006) and disease duration (r = 0.31, p = 0.02), we did not observe significant correlations for the pooled PPMI (n = 19) and BioFIND subjects (n = 38). Note, neurofilament light chain (NfL) data is not available for BioFIND subjects.
